# Cognitive function and metabolic syndrome in unipolar and bipolar depression: A pilot study

**DOI:** 10.1192/j.eurpsy.2021.246

**Published:** 2021-08-13

**Authors:** T. Jannini, L. Longo, F. Marasco, M. Di Civita, C. Niolu, A. Siracusano, G. Di Lorenzo

**Affiliations:** Department Of Systems Medicine, University of Rome Tor Vergata, Rome, Italy

**Keywords:** Metabolic syndrome, cognitive function, bipolar disorder, major depressive disorder

## Abstract

**Introduction:**

Cognitive function is impaired in depressive disorders. Among several factors implicated in regulation of the cognitive function, metabolic syndrome has been showed have a pivotal role cognitive functioning in healthy controls. However, the role of metabolic syndrome in regulating the cognitive functioning of subjects affected by depressive disorders is little studied.

**Objectives:**

To investigate the effect of metabolic syndrome in regulation of cognition in unipolar and bipolar depression.

**Methods:**

One-hundred-sixty-five people affected by a depressive disorder (unipolar depression, UP; bipolar depression, BP) were enrolled at the Psychiatric and Clinic Psychology Unit of the University of Rome Tor Vergata, Rome, Italy. A group of healthy controls (HC) matched for agender and age was enrolled. The cognitive functions were evaluated with a computerized tool, THINC-it.

**Results:**

UP and BP had lower performances in THINC-it cognitive domains than HC. Metabolic syndrome is a negative, independent predictor of low performance in the THINC-it cognitive domains of people with depressive disorders.

**Conclusions:**

Our findings confirm that metabolic syndrome has a prominent role in determining the cognitive efficiency in depressive disorders, independently by the presence of a unipolar or bipolar depressive disorder. Metabolic syndrome has to be considered a major factor that should be considered in the treatment strategies of cognitive functioning improvement of people affected by depressive disorders.

**Disclosure:**

No significant relationships.

